# DNA-inspired nanomaterials for enhanced endosomal escape

**DOI:** 10.1073/pnas.2104511118

**Published:** 2021-05-03

**Authors:** Jinhyung Lee, Ian Sands, Wuxia Zhang, Libo Zhou, Yupeng Chen

**Affiliations:** ^a^Department of Biomedical Engineering, University of Connecticut, Storrs, CT 06269

**Keywords:** DNA nanotechnology, Janus base nanotubes, Janus base nanopieces, endosomal escape, RNA delivery

## Abstract

To realize RNA interference (RNAi) therapeutics, it is necessary to deliver therapeutic RNAs (such as small interfering RNA or siRNA) into cell cytoplasm. A major challenge of RNAi therapeutics is the endosomal entrapment of the delivered siRNA. In this study, we developed a family of delivery vehicles called Janus base nanopieces (NPs). They are rod-shaped nanoparticles formed by bundles of Janus base nanotubes (JBNTs) with RNA cargoes incorporated inside via charge interactions. JBNTs are formed by noncovalent interactions of small molecules consisting of a base component mimicking DNA bases and an amino acid side chain. NPs presented many advantages over conventional delivery materials. NPs efficiently entered cells via macropinocytosis similar to lipid nanoparticles while presenting much better endosomal escape ability than lipid nanoparticles; NPs escaped from endosomes via a “proton sponge” effect similar to cationic polymers while presenting significant lower cytotoxicity compared to polymers and lipids due to their noncovalent structures and DNA-mimicking chemistry. In a proof-of-concept experiment, we have shown that NPs are promising candidates for antiviral delivery applications, which may be used for conditions such as COVID-19 in the future.

For successful RNA interference (RNAi) therapy, it is necessary to deliver RNA cargo to the cytoplasm and escape from late endosomes before degradation (1, 2). Currently, lipid nanoparticles (LNPs) and cationic polymers are the most commonly used vehicles for RNAi delivery. However, LNPs reported a poor endosomal escape ability so a significant amount of RNA cargos were nonfunctional (3, 4). Cationic polymers can escape from endosomes via the “proton sponge” effect, but they are covalently linked and usually have low biodegradability and high cytotoxicity (5, 6). These limitations make it significantly challenging to have a high efficacy of RNAi therapy and impede its translation into clinics (2). Herein, we developed nanopieces (NPs) based on DNA-inspired Janus base nanotubes (JBNTs) for RNAi delivery (Fig. 1*A*). The NPs have chemical compositions from current delivery vehicles and combined advantages in endosomal escape, low toxicity, and high efficacy.

**Fig. 1.**
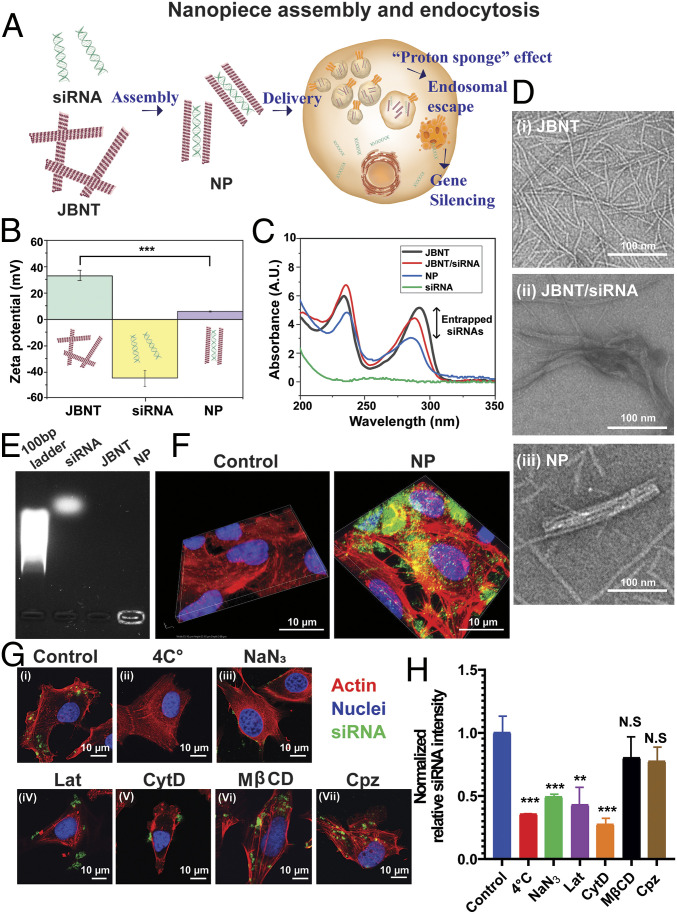
NP assembly and endocytosis. (*A*) Schematic drawing of NPs’ delivery. (*B*) The ζ-potential analysis. (*C*) UV-Vis analysis. (*D*) TEM characterization of the NPs. (*E*) Gel retardation assay. (*F*) CLSM *z*-stack images of siRNA-AF488 (green) delivered by the NPs; cell nuclei stained with DAPI (blue); cell skeleton stained with rhodamine phalloidin (red). (*G*) Inhibition of NP uptake. (*H*) Quantitative analysis of NP uptake. The values are mean ± SEM (*n* ≥ 10). *P < 0.05, **P < 0.01, and ***P < 0.001 compared to control.

## Results

JBNTs are self-assembled from a library of small molecule units (molecular weight, <400 Da). These units consist of two components: a base component mimicking DNA bases and an amino acid side chain. In water, they can assemble into noncovalent nanotubes based on hydrogen bonds and π–π interactions. In this study, a JBNT formula with guanine (G) and cytosine (C) and a lysine side chain was selected. It can incorporate small interfering RNA (siRNA) based on positive–negative charge interactions and base stacking (Fig. 1 *B* and *C*). The ζ potential measurements of siRNA, JBNT, and JBNT/siRNA demonstrated the shift of their surface charge (Fig. 1*B*). UV-visible (Vis) spectra demonstrated there is molecular-level incorporation between JBNTs and siRNA (Fig. 1*C*). When assembled with siRNA, the 280-nm peak of JBNT decreased due to the noncovalent base stacking between JBNT units and siRNA.

After siRNA loading, we can further process JBNT/siRNA into delivery vehicles. JBNTs alone present nanotubular morphology (Fig. 1 *D*, *i*), and they form bundles after incorporating siRNA cargos (Fig. 1 *D*, *ii*). Interestingly, a simple sonication process can break these bundles into smaller individual rod-like vehicles (named Janus base NPs, Fig. 1 *D*, *iii*). The NP’s length is 204.8 ± 14.1 nm, and width is 41.7 ± 4.2 nm. Although the whole NP architecture is formed by noncovalent interactions of their small-molecule units and RNA cargos, the NPs are stable entities. As shown in Fig. 1*E*, NPs encapsulated siRNA cargos and retarded their migration in electrophoresis.

We demonstrated the intracellular delivery ability of the Janus base NPs via confocal laser-scanning microscopy (CLSM) images. Results showed that fluorescently labeled siRNA (green) successfully delivered to cells (Fig. 1*F*). Moreover, because the NPs have chemical compositions different from conventional delivery materials, we also determined their cellular uptake mechanism. We pretreated cells at 4°C or with an ATP inhibitor (NaN_3_) (Fig. 1 *G*, *ii* and *iii*). Results showed that the uptake was significant inhibited by low temperature or ATP inhibition. Therefore, the intracellular delivery of the NPs is energy dependent. To further identity the uptake mechanism, we treated cells with several endocytic inhibitors, including two types of macropinocytosis inhibitors (latrunculin A [Lat]; cytochalasin D [CytD]), a clathrin-mediated inhibitor (chlorpromazine [Cpz]), and a caveolae-mediated inhibitor (methyl-β-cyclodextrin [Mβcd]). Qualitative results in Fig. 1 *G*, *iv*–*vii*, and quantitative results in Fig. 1*H* demonstrated significant inhibition of uptake using macropinocytosis inhibitors.

**Fig. 2.**
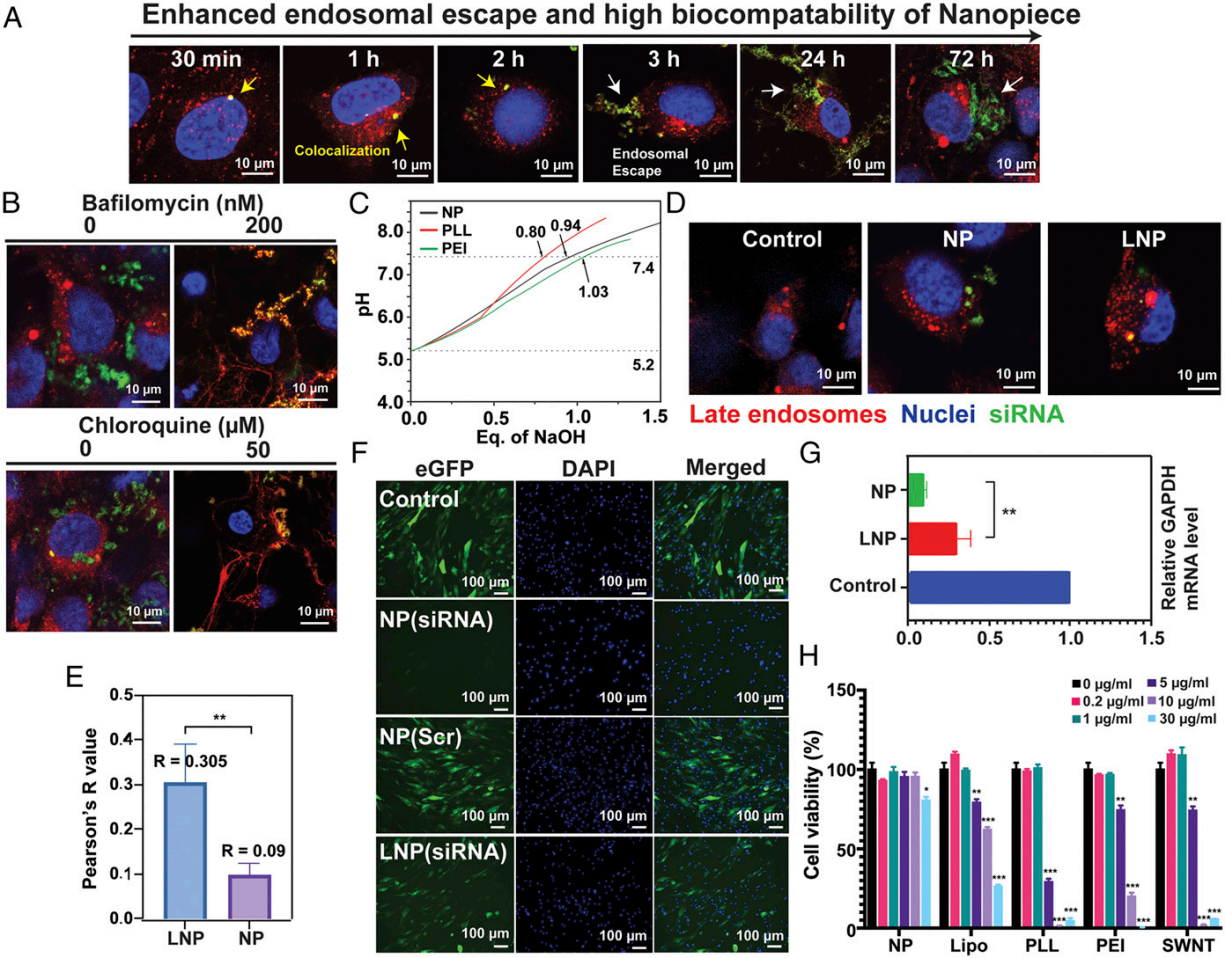
Enhanced endosomal escape and high biocompatibility of the NPs. (*A*) Time-dependent endosomal escape of the NPs. (*B*) Inhibition of the proton sponge effect. (*C*) Acid–base titration curves. (*D*) Endosomal escape of the NPs and LNPs. (*E*) Quantification of colocalization. (*F*) Antiviral ability of NP delivery. (*G*) Inhibition of gene expression of the NPs and LNPs. (*H*) Cell viability analysis. The data were expressed as the percentage of surviving cells, and the values are mean ± SEM (*n* ≥ 10). *P < 0.05, **P < 0.01, and ***P < 0.001 compared to untreated control.

Further assessment of endosomal escape ability of the NPs was conducted. First of all, the kinetics of the endosomal escape was evaluated. As shown in Fig. 2*A*, there is a colocalization (shown as yellow) of siRNA (green) and endosome (red) soon after the delivery (<2 h), demonstrating the siRNA/NPs were uptaken. Then, the NP delivered siRNA was able to escape from endosomes in a time-dependent fashion, showing the differentiation between green and red signals (Fig. 2*A* and Movie S1). Moreover, we identified the mechanism by which the NPs can escape from endosomes. As shown in Fig. 2*B*, the endosomal escape of the NPs was significantly inhibited in the presence of endosomal acidification inhibitors (bafilomycin A1 and chloroquine), so the proton pump is necessary for endosomal escape. Furthermore, we conducted an acid-based titration experiment to assess the buffering capacity of the NPs between pH 7.4 and 5.2 (mimicking pH shift from extracellular to intracellular environment). Results suggested that the NPs have excellent buffering capacity comparable to cationic polymers (such as poly-l-lysine [PLL] and polyethyleneimine [PEI]) (Fig. 2*C*). Therefore, NPs can successfully escape from endosomal entrapments through the “proton sponge” effect.

As mentioned earlier, both Janus base NPs and LNPs were delivered into cells via macropinocytosis into endosomes. The NPs presented much better endosomal escape ability compared to LNPs (Fig. 2*D*). Furthermore, we used Pearson’s *R* values to quantify the colocalization of siRNA and endosome fluorescence. Results have demonstrated significantly lower colocalization of the NPs (*R* = 0.09) compared to LNPs (*R* = 0.305) (Fig. 2*E*).

To evaluate the gene silencing outcomes, we delivered siRNA to inhibit GAPDH (a housekeeping gene) by the NPs or LNPs. Results showed the NPs achieve a better inhibition efficacy than LNPs (maybe due to their enhanced endosomal escape ability) (Fig. 2*G*). Moreover, in response to COVID pandemic, we demonstrated the antiviral potential of the NP delivery of siRNA. Human lung fibroblasts were infected with RGD-fiber modified adenovirus to express enhanced green fluorescent protein (eGFP). The NPs delivered eGFP siRNA successfully inhibited the viral gene expression in these cells (Fig. 2*F*). Again, the NP delivery showed better inhibition efficacy than LNPs.

Finally, we evaluated the cytotoxicity of the NPs. Compared with various commonly used delivery materials: LNPs, cationic polymers (PLL and PEI), and single-walled carbon nanotubes, the Janus base NPs showed dramatically better cell viability (Fig. 2*H*).

## Discussion

Here, we introduced a delivery platform based on DNA-inspired Janus bases. We have built a library of Janus bases. In this study, we used a JBNT with a G^C base with a lysine side chain while we also found JBNTs with adenine (A)^thymine (T) bases or arginine side chains can also effectively deliver small RNAs into cells. Furthermore, we applied a simple sonication approach after JBNTs assembled with RNA cargos to fabricate NPs. Different from conventional spherical nanoparticles, the NPs present a rod shape, so they are long enough for cargo loading as well as slim enough for efficient intracellular delivery. Therefore, we have realized a family of delivery vehicles with chemical compositions different from conventional delivery materials.

LNPs can deliver into various types of cells and the cell uptake mechanism is macropinocytosis, a type of endocytosis (4, 7). In this study, we found the Janus base NPs were also internalized by cells via macropinocytosis. This may explain why the NPs can be internalized by cells efficiently. After endocytosis, LNPs have a poor ability to escape from endosomes while the NPs can escape from endosomes via a proton sponge effect similar to cationic polymers (3, 4, 8). Many polymeric materials have been used for RNAi delivery, but all polymers are formed by covalent bonds (5). As a contrast, the NPs are formed by noncovalent interactions of the Janus base units. The excellent endosomal escape results in this study enlightened a strategy in materials design that noncovalent structures can also achieve excellent proton-sponge capacity similar to covalent polymers.

Cytotoxicity is another very important factor for RNAi delivery. Lipids often produce a proinflammatory response while polymers can be toxic due to poor biodegradability (9, 10). Although the NPs can deliver into cells similar to LNPs and escape from endosomes similar to cationic polymers, they presented much better biocompatibility than lipids, polymers, and carbon nanotubes. This is most likely due to the noncovalent structures and DNA-mimicking chemistry of their Janus bases.

As a study focusing on materials development and mechanism characterizations, we conducted proof-of-concept experiments demonstrating that the NP delivery can result in more effective RNAi in cells compared with conventional methods. For example, in response to the pandemic, we showed that the NPs can successfully inhibit viral gene expression in human lung fibroblasts. Further development is required to engineer an RNAi therapy against SARS-CoV-2 virus or other diseases. As a summary, we have developed a class of delivery vehicles that can achieve intracellular delivery with high efficiency, enhanced endosome escape, and low cytotoxicity.

## Materials and Methods

### Fabrication of NPs.

The G^C units of JBNTs were synthesized as in ref. 11 and the A^T units were synthesized as in ref. 12. The molar ratio between nitrogenous base and amino acid in JBNTs is 1:1. The JBNT/siRNA were assembled by mixing JBNT and siRNA (1:10 molar ratio) in nuclease-free water, followed by sonicated with Sonicator (Q Sonica; Sonicators) at 100% amplitude for 2 min and 30 s.

### Characterization of NPs.

The particles and ζ potential of the NPs were measured by dynamic light scattering (Zetasizer; Malvern Panalytical), and the morphology was observed by transmission electron microscope (TEM) (Tecnai T12). The gel retardation assay was conducted at 0.8% low-melting agarose gel followed by electrophoresis at 100 V for 50 min. The UV-Vis absorption spectra were recorded for each solution with a NanoDrop One/One^c^ (Thermo Fisher Scientific). For the measurement of buffering capacity of NPs and polymers, NP and cationic polymers at the same 0.08 μmol were titrated by either adding the 2 μL of 10 mM HCl or 10 mM NaOH.

### NP Delivery.

Assembled NPs were immediately transferred to C28/I2 cells and then incubated at 37 °C and 5% CO_2_. Then, cells were fixed with 4% formaldehyde, treated with Triton X, and stained with rhodamine phalloidin (30 min) and DAPI (10 min). A Nikon A1 confocal laser-scanning microscope was used for fluorescence imaging.

For cell uptake mechanism studies, cells were exposed to several different concentrations of the inhibitors for 1 h, pretreated with Cpz hydrochloride (100 μM for 30 min), Mβcd (1 mM for 30 min), CytD (4 μM for 1 h), Lat (2 μM for 30 min), bafilomycin A1 (200 nM for 30 min), and chloroquine (10 μM for 30 min).

For endosomal escape studies, Lysotracker Red DND-99 (catalog #L7528; Invitrogen) was used before fixing (instead of rhodamine). The degree of colocalization was quantified based upon Pearson’s correlation coefficient (*R*) using ImageJ software following the colocalization threshold and coloc2 plugin.

For the siRNA knockdown study, NPs was used to deliver GAPDH siRNA for 24 h. Lipofectamine 2000 was used as a control according to the manufacturer’s protocol. The gene expression was analyzed by RT-PCR. According to manufacturer’s protocol, TRIzol reagent (Bio-Rad), iScript cDNA synthesis kit (Bio-Rad), and SYBR Green master mix for qPCR (Bio-Rad) were used to isolate, reverse transcribe, and measure mRNA expression. Data were normalized by 18S ribosomal RNA.

### Antiviral Study.

GFP expressing RGD fiber modified Adenovirus (Vector BioLabs) was pretreated (multiplicity of infection, 10) to the human lung fibroblast cells in each well following the steps on the product manual. NPs or LNP containing the Silencer eGFP siRNA (Thermo Fisher) were transfected for 24 h. A fluorescence microscope (ZOE Fluorescent Cell imager) was used to take the cell images.

### Cell Toxicity Assay.

The cell viability of various vectors, including NP, were determined by CKK-8 assay (Sigma Cell Counting Kit-8). After coincubation for 24 h, the absorbance was obtained by a microplate reader (Molecular Devices).

### Statistics.

Statistical analyses were performed by using GraphPad Prism 7 software. Error bars were expressed as the mean ± SEM. Numerical data were analyzed via Student’s *t* test, followed by ANOVA.

## Supplementary Material

Supplementary File

Supplementary File

## Data Availability

All study data are included in the article and/or supporting information.
