# Supplementary material for: DNA-inspired nanomaterials for enhanced endosomal escape
Source: Proc Natl Acad Sci U S A. 2021 May 3;118(19):e2104511118. doi: 10.1073/pnas.2104511118 (PMC8126792; doi:10.1073/pnas.2104511118)
Supplement: Supplementary File [file pnas.2104511118.sapp.pdf]

PNAS
www.pnas.org

Supplementary Information for
DNA-inspired nanomaterials for enhanced endosomal escape

[1]Jinhyung Lee, [1]Ian Sands, [1]Wuxia Zhang, [1]Libo Zhou, and [1]Yupeng Chen*
[1]Department of Biomedical Engineering, University of Connecticut, Storrs, Connecticut 06269
*Corresponding author: yupeng.chen@uconn.edu

Movie S1. 3D movie of the endosomal escape of the NPs: Z-stack CLSM images of NP delivered AF488-siRNA (**green**) escaping late endosomes (**red**), (cell nuclei were stained in **blue**).
